# Increased Risk of Lateral Femoral Cutaneous Nerve Injury in Patients With Previous Hip Arthroscopy Who Underwent a Direct Anterior Approach Total Hip Arthroplasty

**DOI:** 10.1016/j.asmr.2022.10.013

**Published:** 2022-12-07

**Authors:** Adam S. Gerry, Jose M. Iturregui, Brian J. Carlson, Jeffrey D. Hassebrock, Zachary K. Christopher, Mark J. Spangehl, Kostas J. Economopoulos, Joshua S. Bingham

**Affiliations:** a Department of Orthopedics, Mayo Clinic Arizona, Phoenix, Arizona; b Midwestern University, AZCOM, Glendale, Arizona; c Elson S. Floyd College of Medicine, Washington State University, Spokane, Washington, USA

## Abstract

**Purpose:**

To evaluate the rates of lateral femoral cutaneous nerve (LFCN) injury in patients who underwent a direct anterior approach (DAA) total hip arthroplasty (THA) with and without previous hip arthroscopy.

**Methods:**

We retrospectively investigated consecutive DAA THAs performed by a single surgeon. These cases were grouped into patients with and without a history of previous ipsilateral hip arthroscopy. LFCN sensation was assessed during the initial follow-up (6 weeks) and 1-year (or most recent) follow-up visits. The incidence and character of LFCN injury was compared between the 2 groups.

**Results:**

In total, 166 patients underwent a DAA THA with no previous hip arthroscopy, and 13 had a history of previous arthroscopy. Of the 179 total patients who underwent THA, 77 experienced some form of LFCN injury at initial follow-up (43%). The rate of injury for the cohort with no previous arthroscopy was 39% (n = 65/166) on initial follow-up, whereas the rate of injury for the cohort with a history of previous ipsilateral arthroscopy was 92% (n =12/13) on initial follow-up (*P* < .001). In addition, although the difference was not significant, 28% (n = 46/166) of the group without history of previous arthroscopy and 69% (n = 9/13) of the group with a history of previous arthroscopy had continued symptoms of LFCN injury at most recent follow-up.

**Conclusions:**

In this study, patients who underwent hip arthroscopy before an ipsilateral DAA THA were at increased risk of LFCN injury compared with patients who underwent a DAA THA without a previous hip arthroscopy. At final follow-up of patients with initial LFCN injury, symptoms resolved in 29% (n = 19/65) of patients with no previous hip arthroscopy and 25% (n = 3/12) of patients with previous hip arthroscopy.

**Level of Evidence:**

Level III, case–control study.

Total hip arthroplasty (THA) is one of the most successful and commonly performed orthopaedic procedures. Current data suggest that THA is performed on more than 468,000 patients annually in the United States,^1^ and the rate of primary total hip arthroplasties has increased in the United States by approximately 50% over the 13-year span between 1990 and 2002.^2^ Evidence from 2010 showed the prevalence of THA in the total U.S. population to be 0.83%, accounting for 2.5 million individuals, with trends indicating a rise in prevalence over time.^3^ Based on current trends, it is estimated that the volume of THA procedures will increase to 635,000 per year by the year 2030.^4^ Additionally, Liu et al.^5^ showed that over a 15-year study period, patients undergoing THA have had a decrease in average age toward the younger than 65 years old category. Generally, THA is a successful procedure, with good clinical outcomes, a low risk of revision, and 20-year survival rates up to 85%.^6^ Patient satisfaction following the procedure is also quite high, with reported patient satisfaction following THA in up to 91% of cases.^7^, ^8^, ^9^

Recently, the direct anterior approach (DAA) to THA has grown in popularity because of the purported benefits of early restoration of gait kinematics, quick recovery time, and lower dislocation rates.^5^^,^^10^, ^11^, ^12^, ^13^ One notable downside to this approach is the risk of injury to the lateral femoral cutaneous nerve (LFCN) and its branches. The branching pattern of the LFCN varies in individuals and can course anywhere from 6.5 cm medial to 6 cm lateral to the anterior superior iliac spine (ASIS).^14^ Studies in cadavers have shown that as a result of these variations, 33% to 42% of patients are at risk of LFCN injury during standard DAA approaches for THA.^15^^,^^16^

The actual incidence of LFCN injury reported ranges from 12% to 81% in the literature.^17^, ^18^, ^19^, ^20^ Although these data are concerning, most studies show that the majority of neuropraxic injuries tend to resolve without any long-term functional sequelae. Goulding et al.^19^ demonstrated that in 132 patients with LFCN neuropraxia, pain scores were minimal, and no functional impairments were identified at a mean follow-up of 13 months. Further studies show LFCN complications following DAA THA ranging from 12%^21^ to 37%^18^ of patients, with both studies showing no difference in functional outcomes. Regardless of the lack of functional deficits or limitations, these LFCN injuries can still cause significant discomfort and affect quality of life for patients.

There are several known risk factors for LFCN injury that have been described in the literature, including autograft harvesting from the iliac crest, periacetabular osteotomies, acetabular fixation, and posterior thoracolumbar spine surgery.^19^^,^^22^, ^23^, ^24^, ^25^ One potential risk factor that has not been well defined is a history of previous ipsilateral hip arthroscopy. Patients who undergo hip arthroscopy carry an increased risk of LFCN injury of anywhere from 0.3% to 2%, as reported in literature.^26^, ^27^, ^28^, ^29^ A portion of patients who undergo hip arthroscopy ultimately progress to THA in the years following their hip arthroscopies. A review of 7,351 patients who underwent hip arthroscopy with 2 years of follow-up showed that 12% of those patients underwent conversion to THA within the 2 years following hip arthroscopy.^30^ Currently, it is unclear whether patients are at an increased risk of LFCN injury after undergoing both procedures. Therefore, the purpose of this study was to evaluate rates of LFCN injury in patients who underwent a DAA THA with and without previous hip arthroscopy. We hypothesized that patients with a history of hip arthroscopy would be at an increased risk of LFCN injury following the DAA.

## Methods

This study was deemed exempt by the institutional review board. We retrospectively investigated consecutive DAA hip arthroplasty operations performed by a single fellowship-trained arthroplasty surgeon (J.S.B.) from 2019 through 2021. Of the 179 total patients, all were included in our study. These cases were grouped by history of previous hip arthroscopy (Table 1). One group was characterized by a history of no previous hip arthroscopy (NPHA) and the second one was characterized by a history of previous hip arthroscopy (PHA). In general, hip arthroscopy consisted of 3 portals: a lateral portal as the main viewing portal, an anterior portal, and a mid-anterior portal. The lateral portal is typically made just superior to the greater trochanter, the anterior portal is made a few centimeters medial to this, just medial to the plane of the ASIS, and finally the mid-anterior portal is created directly between the lateral and anterior portals approximately 3 to 5 cm distal. LFCN sensation was assessed during the initial follow-up (6 weeks) and 1-year (or most recent) follow-up visits.

**Table 1:** Overall Patient Characteristics

Demographics	No Previous Hip Arthroscopy (n = 166)	Previous Hip Arthroscopy (n = 13)	*P* Value
Age, y	65.6 ± 10.7	49.7 ± 11	<.0001 ∗
Sex (% male)	43.9% (n = 73)	53.9% (n = 7)	.49
BMI	26.8 ± 3.8	26 ± 3.5	.49
ASA class	2.1 ± 0.58	1.9 ± 0.55	.15
Time between most recent arthroscopy and arthroplasty, mo (range)	N/A	38.9 (9-156)	N/A
Average follow-up, mo (range)	22.0 (2-53)	15.8 (2-38)	.036 ∗

NOTE. Compared are age, sex, BMI, and ASA class between the no previous arthroscopy and previous hip arthroscopy groups to determine significant differences in averages.

ASA, American Society of Anesthesiologists; BMI, body mass index; N/A, not available.

### Surgical Approach and Closure

All patients were appropriately positioned on the Hana table. A 7-cm long incision based 2 cm lateral and 1 cm distal to the ASIS was used, extending distally in line with the femoral shaft. The fascia overlying the tensor fascia lata (TFL) was incised in line with its fibers lateral to the sartorial/tensor plane to avoid LFCN injury. Care is taken at this point to preserve any visible branches of the LFCN when possible. The fascia was then elevated above the TFL and the standard Hueter approach was used. The lateral circumflex artery was ligated and the investing fascia over the capsule was divided and preserved. The femoral neck was osteotomized and removed. The acetabulum was reamed, and the acetabular shell was press fit and supplemented with supplemental cancellous bone screws.

Next, the leg was externally rotated, and the femoral canal was broached. A new femoral head was placed, and the hip was reduced and moved through appropriate range of motion. After confirmation of trial implants, the permanent femoral head was impacted into place. The wound was thoroughly irrigated, the capsule was closed, and the fascia over the TFL was closed with running sutures, with care taken to avoid wrapping up any branches of the LFCN during fascial closure.

There is no validated diagnostic tool for LFCN neuropraxia, so sensation was collected during the initial and 1-year (or most recent) follow-up patient encounters. Patients were asked whether they felt any dysesthesias on the operative leg, including numbness, tingling, burning, or pain during the physical examination. Numbness overlying or directly adjacent to the incision was considered peri-incisional numbness and not considered LFCN neuropraxia. We defined LFCN injury as the presence of any of these previously mentioned symptoms over the lateral aspect of the thigh.

To evaluate the significance of a previous history of hip arthroscopy before DAA THA in evaluating patients with LFCN nerve injury, a χ^2^ analysis was performed on the 179 patients and the NPHA versus PHA subgroups in our sample. Broadly, we analyzed the data to investigate whether age, body mass index (BMI), sex, or the American Society of Anesthesiologists (ASA) score had any significance in groups who had LFCN injury versus those who did not using 2-tailed *t*-tests with significance of *P* < .05. Determining the significance in difference between averages in specific demographic determinants was performed using a 2-tailed *t*-test with a significance level of *P* < .05. All statistical calculations were performed using SPSS statistical software, version 28 (IBM Corp., Armonk, NY).

## Results

A total of 179 patients were included in the study (Table 1). The average age of the NPHA group was 66 years and was 44% male. The average age of the PHA group was 50 years and was 54% male. The PHA group was significantly younger than the NPHA group (*P* < .0001). In total, the entire cohort had a mean follow-up of 21.8 months (range, 2-53 months). A total of 166 patients underwent a DAA THA with no previous hip arthroscopy and 13 had a previous arthroscopy (Table 1). In the PHA group, 2 patients had a history of multiple arthroscopies before undergoing THA. Every patient included in the study underwent a similar surgical approach.

Thirteen patients had a hip arthroscopy ranging from 9 months to 13 years before THA. The average duration between hip arthroscopy and arthroplasty was 39 months (Table 1). Of the 179 patients who underwent THA, 77 experienced some form of LFCN injury at initial follow-up postsurgery. LFCN injury was significantly lower at 39% in the DAA THA group with no previous arthroscopy compared with 92% in the group with a previous hip arthroscopy (*P* < .001) (Table 2). There was no significant difference in BMI or ASA class in incidence of LFCN injury at initial follow-up. There was a significant difference in age between the PHA and NPHA groups, with the PHA group being younger by an average of 15 years (*P* < .001).

**Table 2:** Characteristics of Patients with LFCN Injury at Initial Follow-up

Demographics	No Previous Hip Arthroscopy (n = 65)	Previous Hip Arthroscopy (n = 12)	*P* Value (Significant if <.05)
Percentage of initial group	39.2% (n = 65/166)	92.3% (n = 12/13)	<.001∗
Age, y	64.1 ± 11.4	49.7 ± 11	<0.001∗
Sex (% male)	53.9% (n = 35)	58.3% (n = 7)	.78
BMI	26.7 ± 3.9	26 ± 3.5	.38
ASA class	2.1 ± 0.59	1.8 ± 0.57	.19

NOTE. χ^2^ values for “percentage of initial group” show that the incidence of LFCN injury (general) was greater in the previous hip arthroscopy group. Two-sided *t*-tests were used comparing age, sex, BMI, and ASA in those with LFCN injury at initial follow-up between the no previous arthroscopy and previous hip arthroscopy groups to determine significant difference in averages.

ASA, American Society of Anesthesiologists; BMI, body mass index; LFCN, lateral femoral cutaneous nerve; N/A, not available.

Of the patients who continued to show LFCN injury symptoms on most recent follow-up, sex, BMI, and ASA did not show significant differences between the 2 groups. However, there was a significant difference in age between the 2 groups with the previous arthroscopy group with continued symptoms being younger by an average of 14 years (*P* < .001) (Table 3).

**Table 3:** Characteristics of Patients With LFCN Injury at Most Recent Follow-up

Demographics	No Previous Hip Arthroscopy (n = 46)	Previous Hip Arthroscopy (n = 9)	*P* Value (Significant if <.05)
Age, y	64.1 ± 9.8	50.5 ± 11.9	<.001∗
Sex (% male)	60.9% (n = 28)	77.8% (n = 7)	.75
BMI	26.7 ± 4.1	24.5 ± 2.8	.14
ASA class	2.2 ± 0.57	1.8 ± 0.44	.05

NOTE. Two-sided *t*-tests were used comparing age, sex, BMI, and ASA in those with LFCN injury at most-recent follow-up between the no previous arthroscopy and previous hip arthroscopy groups to determine significant difference in averages.

ASA, American Society of Anesthesiologists; BMI, body mass index; LFCN, lateral femoral cutaneous nerve; N/A, not available.

The type of the LFCN symptoms were further characterized as numbness, tingling, pain, and burning (Table 4). Numbness was present in all cases of LFCN injury. Symptoms of tingling significantly decreased from initial to final follow-up in both groups (*P* < .003). There were no patients in either group who reported pain at most recent follow-up. The sensations of pain that were present in 2 patients in the PHA group and 1 patient in the NPHA group at the initial follow-up had resolved by the most recent follow-up visit.

**Table 4:** LFCN Injury Specific Complications and Comparison

Complications	Initial Follow-Up			Most Recent Follow-Up		
	Previous Hip Arthroscopy	No Previous Hip Arthroscopy	*P* Value	Previous Hip Arthroscopy	No Previous Hip Arthroscopy	*P* Value
Numbness	n = 12 (100%)	n = 65 (100%)	*P* < .001∗	n = 9 (100%)	n = 46 (100%)	*P* < .003∗
Tingling	n = 6 (50%)	n = 10 (15.4%)	*P* < .00001∗	n = 4 (44.4%)	n = 1 (2.2%)	*P* < .05∗
Pain	n = 2 (16.7%)	n = 1 (1.5%)	*P* < .05∗	n = 0	n = 0	
Burning	n = 1 (8.3%)	n = 0 (0%)		n = 0	n = 0	

NOTE. Incidences of specific complications within the “previous arthroscopy” and “no previous arthroscopy” groups. For groups that had patients with 0 incidence of injury, *P* values could not be calculated.

When we evaluated the 179 total patients for risk factors (without taking PHA vs NPHA into consideration), BMI and ASA were not significant risk factors for LFCN injury following DAA THA (*P* = .46 for BMI and *P* = .5 for ASA). Age, however, did have a significant impact on LFCN injury (*P* = .004), with the group who presented with LFCN injury being younger on average than the group who presented without initial LFCN injury. In addition, sex had a significantly different incidence in LFCN injury versus those without (*P* = .02), with a greater rate of LFCN injuries in males.

When analyzing improvement in symptoms from initial to most recent follow-up (Table 5), 69% of patients with a previous hip arthroscopy continued to have symptoms, including numbness and tingling, whereas only 28% of patients with no previous hip arthroscopy had continued symptoms. However, this difference did not reach significance when we used χ^2^ analysis (*P* = .77).

**Table 5:** Improvements in LFCN Injury Symptoms Between Initial and 1-Year (or Most Recent) Follow-Up

	Initial Follow-up	Most Recent Follow-up	Percentage of Patients With Improved Symptoms
Previous arthroscopy	92.3% (12/13)	69.2% (9/13)	25% (3/12)
No previous arthroscopy	39.1% (65/166)	27.7% (46/166)	29.2% (19/65)

NOTE. Using χ^2^ analysis, *P* = .77; there is not a significant difference in percentage of patients with improved symptoms between the 2 groups.

Of the 22 patients who showed improvement (had a complaint of either numbness, tingling, burning, or pain initially with no complaints at most recent follow-up), qualifiers including BMI (*P* = .65), age (*P* = .82), and ASA scores (*P* = .10) did not show significant difference versus patients who had continued symptoms. However, sex differences did show significance (*P* = .04) in patients who showed improvement, where female patients were more prevalent in the group that showed resolution of symptoms and male patients more prevalent in the group that had continued symptoms.

## Discussion

In this cohort, patients with a previous hip arthroscopy were at significantly increased risk of initial LFCN injury after undergoing a subsequent DAA THA (39% in NPHA vs 92% in PHA). We hypothesize that this increased rate of LFCN injury may be attributable to additional traction placed on the nerve secondary to scar tissue associated with the previous hip arthroscopy. Orthopaedic surgeons should be aware of these risks when performing DAA hip replacements on patients who have had a previous hip arthroscopy, and patients should be educated about this risk. Younger age also was found to be a risk factor for LFCN injury following DAA hip arthroplasty in our study. However, this finding may be related to the fact that the cohort of patients undergoing previous hip arthroscopy before hip arthroplasty was significantly younger than the patients who did not have previous hip arthroscopy (*P* < .0001). In general, hip arthroscopy is reserved for younger patients with less arthritis. Although age itself may be an independent risk factor for LFCN injury following DAA hip arthroplasty, the association with younger age may be secondary to the fact that younger patients are more prone to undergo hip arthroscopy, which may be the true risk factor.

In addition, our data showed that the LFCN injury following DAA THA was more prevalent in male patients. Furthermore, there was a significant difference in rates of improvement of symptoms of LFCN injury with better improvement associated with female patients. Rudin et al.^15^ describe the several different types of branching patterns of the LFCN and state that approximately one third of patients have a fan-type LFCN distribution and that this anatomical course of the nerve is especially at risk in anterior-approach hip surgery. Studies have yet to determine whether this fan-type LFCN distribution is more common in the male sex, but these or other anatomic differences could be a plausible explanation for our findings that LFCN injury was more common in males.

Our data also demonstrate that almost 70% of patients with a previous arthroscopy who had a LFCN neuropraxia were still experiencing numbness at the most recent follow-up (Table 5). Regardless of functional impact postoperatively, these symptoms can affect the quality of life of patients. Although no patients in this group required further intervention, treatment options for nerve irritation and injury should be explored. While most authors recommend conservative treatment options, treatment of meralgia paresthetica with ultrasound-guided perineural methylprednisolone has shown substantial symptom relief.^31^^,^^32^ Studies also have shown that treatment with a nerve stimulator while performing regional anesthetic block of the nerve has significantly reduced symptoms of LFCN injury.^31^^,^^33^^,^^34^ Upon failure of all other conservative measures, surgical release of an injured lateral femoral cutaneous nerve has shown increases in patient satisfaction.^35^ These techniques may be an option for treatment of symptomatic LFCN injury post-DAA.

Our overall LFCN injury rate for after DAA (43% on initial follow-up) is within the range of previously reported LFCN injury rates for DAA approaches to THA.^20^ However, the rate of LFCN injury in patients with a previous hip arthroscopy was much greater (92%). Although these injuries are often subjective to the patient and there is no standard classification, these studies support that there are sensory complications after a DAA THA without an accompanying functional limitation. It would be useful to have a standardized classification system for these injuries to gain a deeper understanding of the true prevalence of LFCN injury following DAA. Overall, our data demonstrated that LFCN injury after DAA THA was more common in patients who previously underwent ipsilateral hip arthroscopy. This is important information for surgeons to use in counseling their patients regarding their risk of LFCN injury. Future studies in larger cohorts are necessary to evaluate this complication and its impact.

### Limitations

Limitations to this study include a small sample size, a significant difference in age between groups, a short average follow-up of 21.8 months between date of surgery and most recent visit, and a lack of a validated survey to assess LFCN injury. Furthermore, this is a single-surgeon series and, as such, the incidence may vary on the basis of surgeon and surgical technique. The significant difference in age could be attributed to the fact that most people undergoing hip arthroscopy are younger than those patients who go straight to hip arthroplasty.

## Conclusions

In this study, patients who underwent hip arthroscopy before an ipsilateral DAA THA were at increased risk of LFCN injury compared with patients who underwent a DAA THA without a previous hip arthroscopy. At final follow-up of patients with initial LFCN injury, symptoms resolved in 29% (n = 19/65) of patients with no previous hip arthroscopy and 25% (n = 3/12) of patients with previous hip arthroscopy.

## Supplementary Data

ICMJE author disclosure forms
